# Exploring Patient and Staff Experiences With Video Consultations During COVID-19 in an English Outpatient Care Setting: Secondary Data Analysis of Routinely Collected Feedback Data

**DOI:** 10.2196/30486

**Published:** 2022-03-21

**Authors:** Hannah Bradwell, Rebecca Baines, Katie J Edwards, Sebastian Stevens, Kate Atkinson, Ellen Wilkinson, Arunangsu Chatterjee, Ray B Jones

**Affiliations:** 1 Centre for Health Technology University of Plymouth Plymouth United Kingdom; 2 Cornwall Partnership National Health Service Foundation Trust Cornwall United Kingdom

**Keywords:** COVID-19, video consultations, remote consultation, Attend Anywhere, patient feedback, patient experience, staff feedback, outpatients, pandemic

## Abstract

**Background:**

Video consultations (VCs) were rapidly implemented in response to COVID-19 despite modest progress before.

**Objective:**

We aim to explore staff and patient experiences with VCs implemented during COVID-19 and use feedback insights to support quality improvement and service development.

**Methods:**

Secondary data analysis was conducted on 955 patient and 521 staff responses (from 4234 consultations; 955/4234, 22.6% and 521/4234, 12.3%, respectively) routinely collected following a VC between June and July 2020 in a rural, older adult, and outpatient care setting at a National Health Service Trust. Responses were summarized using descriptive statistics and inductive thematic analysis and presented to Trust stakeholders.

**Results:**

Most patients (890/955, 93.2%) reported having *good* (210/955, 22%) or *very good* (680/955, 71.2%) experience with VCs and felt listened to and understood (904/955, 94.7%). Most patients accessed their VC alone (806/955, 84.4%) except for those aged ≥71 years (23/58, 40%), with ease of joining VCs negatively associated with age (*P*<.001). Despite more difficulties joining, older adults were most likely to be satisfied with the technology (46/58, 79%). Patients and staff generally felt that patients’ needs had been met (860/955, 90.1% and 453/521, 86.9%, respectively), although staff appeared to overestimate patient dissatisfaction with VC outcomes (*P*=.02). Patients (848/955, 88.8%) and staff (419/521, 80.5%) felt able to communicate everything they wanted, although patients were significantly more positive than staff (*P<*.001). Patient satisfaction with communication was positively associated with technical performance satisfaction (*P*<.001). Most staff members (466/521, 89.4%) reported positive (185/521, 35.5%) or very positive (281/521, 53.9%) experiences with joining and managing VCs. Staff reported reductions in carbon footprint (380/521, 72.9%) and time (373/521, 71.6%). Most patients (880/955, 92.1%) would choose VCs again. We identified three themes in responses: barriers, including technological difficulties, patient information, and suitability concerns; potential benefits, including reduced stress, enhanced accessibility, cost, and time savings; and suggested improvements, including trial calls, turning music off, photo uploads, expanding written character limit, supporting other internet browsers, and shared online screens. This routine feedback, including evidence to suggest that patients were more satisfied than clinicians had anticipated, was presented to relevant Trust stakeholders, allowing for improved processes and supporting the development of a business case to inform the Trust decision on continuing VCs beyond COVID-19 restrictions.

**Conclusions:**

The findings highlight the importance of regularly reviewing and responding to routine feedback following digital service implementation. The feedback helped the Trust improve the VC service, challenge clinician-held assumptions about patient experience, and inform future use of VCs. It has focused improvement efforts on patient information; technological improvements such as blurred backgrounds and interactive whiteboards; and responding to the needs of patients with dementia, communication or cognitive impairment, or lack of appropriate technology. These findings have implications for other health care providers.

## Introduction

COVID-19 is a global health concern [1] that has resulted in the rapid implementation and digitalization of many health care services [2,3]. As a result, video consultations (VCs), also referred to as remote or virtual consultations [4], now form an integral part of both primary and outpatient (ambulatory) care. Although limiting viral exposure and reducing the potential risk of infection for patients and staff [4,5], VCs may also enable additional visual cues beyond the capabilities of telephone consultations, helping further facilitate therapeutic relationships and experiences of care [2].

Although there is guidance on how to deliver VCs [6-8] and growing evidence exploring the rapid implementation of VCs in various areas of health care [4,9-17], there are relatively few empirical studies on VCs. For example, Doraiswamy et al [16] reviewed 543 articles related to telehealth (including telephone, VC, and other communication methods) during COVID-19, and only 12% of articles presented empirical work, with few studies conducted in the United Kingdom focusing on VC [16]. Similarly, other research has focused on a single service, such as orthopedics or mental health [4,13,14]. Although the research by Dhahri et al [15] focuses on feedback across a range of outpatient services from 45 patients and 79 clinicians, this was only over a 3-week period.

The emerging research on remote health care and VC implementation seems to show some benefits. For example, VCs are seen as useful for social distancing [12], may provide quicker consultation times [4], reduce travel time for patients [14], and allow for safeguarding and risk assessment [13]. However, the research to date also shows areas of concern, such as technology limitations (impairing video and sound) [4,9,15], additional burden, lack of physical examination [9], low technology confidence and limited setup support [4], and impaired therapeutic interactions and reduced depth of clinical encounters [14].

However, the sample size of VC respondents in studies to date is limited, and no studies to our knowledge have been reported from a British rural and deprived region with an older population [18]. Documented implications of routine feedback on practice have also been lacking in previous literature. Thus, our study aims to investigate National Health Service (NHS) staff and patient experiences with the Attend Anywhere VC in Cornwall using routine feedback from a large sample and to explore the impact of insights shared when presented to key stakeholders in the service.

## Methods

### Design

This study consists of a secondary analysis of routinely collected, anonymized survey data following a VC that was designed and distributed by the partner health care provider (NHS Trust) and subsequent follow-up to disseminate results and assess the impact of feedback with the Trust.

### Setting

The Cornwall Foundation Partnership NHS Trust provides mental health and community services for Cornwall and the Isle of Scilly, a geographically isolated peninsula in the South West of England that experiences higher than average levels of deprivation [19]. By UK definitions, Cornwall and the Isle of Scilly are very rural, with 40% of the population living in remote areas [19], an older age profile [20], and a single acute hospital located in the center of the county. The Trust’s services include children and adolescent mental health, adult mental health, and physical health, including but not limited to learning disabilities, cardiac services, bladder and bowel, complex care and dementia, eating disorders, personality disorders, psychiatric liaison, palliative care, stroke nursing, speech and language therapy, diabetes, epilepsy, minor injuries, musculoskeletal care, neurorehabilitation, physiotherapy, podiatry, and respiratory nursing. This study was carried out by an independent research team using anonymous data provided by the Trust.

### VC Service

The Trust started using Attend Anywhere, a telemedicine platform for outpatient care [21,22], on April 6, 2020. The platform facilitates video calls between clinicians and patients for scheduled appointment times. The video calls can be conducted over internet-connected computers, phones, or tablets. The implementation process followed guidance provided by NHS England and NHS Improvement [23]. Some appointments were still carried out face to face, but many patients were offered VC or telephone contact during the study period.

### Data Collection

The Trust set up a system of routine feedback using a web-based survey (Multimedia Appendix 1) based on the standardized feedback survey questions provided in the national guidance [23]. Immediately after participating in a VC, all staff and patients were invited to complete the web-based survey regardless of whether they had completed one before. The survey was presented via the Meridian surveying platform at the end of a VC. To our knowledge, the survey was not designed with patient or public involvement owing to the rapid implementation process. The rapid rollout of this feedback process may also be responsible for some limitations in the surveys themselves, including limitations in response options and understanding of the sample (eg, number of unique individuals and complete understanding of the service accessed by the patient). However, we have confidence that the data items used in the analysis are robust based on their recommended use by NHS England and NHS Improvement.

### Participants

Participation was voluntary, and the patient participants were patients or their carers. Survey respondents gave consent for their data to be used, but some chose not to have comments publicly shared. Their data were included in the analysis and production of the themes, but their quotes were not included. We used data collected during the early implementation of VCs in response to COVID-19 (June 1, 2020, to July 31, 2020). During this time, 4234 Attend Anywhere appointments were completed. The feedback data used were 22.56% (955/4234) patient and 12.3% (521/4234) staff responses out of the 4234 completed VCs. The sample size was largely pragmatic, using data from as many patients (nearly 1000) as was thought possible to thematically analyze in a timely manner to give feedback to staff during a 2-month period after an initial *settling down* of the system but early enough to have practical use in assessing the utility of the method.

### Data Analysis

Descriptive statistics were reported for numerical data, and chi-square and Mann–Whitney *U* tests were used where appropriate. Free-text responses were analyzed by 4 researchers (HB, RB, KE, and SS) using inductive thematic analysis [24]. Initially, staff and patient comments were analyzed separately. A comprehensive coding framework was developed. Patient and staff codes demonstrated high comparability and, thus, are presented as combined themes across the data set, with areas of discordance discussed. Thematic analysis was selected as a useful and flexible method to generate a rich yet detailed and complex account of qualitative data [24]. Adopting an inductive approach also helped ensure that identified themes arose from the data generated as opposed to predefined concepts or ideas.

### Documented Impact of Routine Feedback on Real-world Practice

Following analysis of the results, summary presentations were created and presented to relevant stakeholders as rapid feedback between November 24, 2020, and February 21, 2021. Presentations were given in partnership with a service-user consultant to patients, patient representatives, and professionals at an Experiences of Care collaborative meeting within the care system, the South West Outpatient Transformation group, the region’s VC forum, and local web-based research dissemination events, and to national audiences through the Outpatient Transformation regional leads meeting. The results were also shared with interested international health care providers (Finland).

### Patient and Public Involvement

The research question and study were informed by patient input (through inclusion of patient experience), and a service-user consultant contributed to reviewing and coauthoring the manuscript.

### Ethics Approval

Ethical approval to conduct this secondary analysis was provided by the Health Research Authority and Health and Care Research Wales, Integrated Research Application System ID286543 (27.07.2020). This manuscript was prepared using the Standards for Quality Improvement Reporting Excellence guidelines [20].

### Data Sharing Statement

Not all patients consented for their comments to be published; thus, the full data set is not publicly available. However, interested parties may inquire with the authors for further details.

## Results

The results are presented in three sections: (1) quantitative results, including participant characteristics; (2) qualitative results; and (3) documented impact of this routine feedback on real-world practice. Multimedia Appendix 1 provides further details on the questions presented to staff and patients in the survey.

### Quantitative Results

#### Patient Age and Device Used

Just under a quarter (955/4234, 22.56%) of the 4234 patient VCs resulted in feedback responses. As the data were anonymous, it was not possible to know if some individuals completed the survey more than once in this 2-month period. Therefore, each data entry was treated as an individual episode. The highest number of survey responses was received from individuals aged 31-50 years (333/955, 34.9%), with the lowest response from patients aged >71 years (58/955, 6.1%). Half of the patients (487/955, 51%) used a laptop to access their VC. Devices used varied by age (χ^2^_12_=68.9; *P*<.001)—patients aged >50 years were much less likely to use mobile devices and more likely to use a tablet (Table 1).

**Table 1:** Use of devices for the video consultation by age group, showing numbers (percentages; N=955).

Item		Device, n (%)				Total, n (%)
		Laptop	Mobile phone	Tablet	Other	
**Age group (years)**						
	<18	82 (50.3)	44 (27)	32 (19.6)	5 (3.1)	163 (17.1)
	18-30	76 (52.1)	35 (24)	24 (16.4)	11 (7.5)	146 (15.3)
	31-50	167 (50.2)	87 (26.1)	64 (19.2)	15 (4.5)	333 (34.9)
	51-71	120 (49.6)	20 (8.3)	70 (28.9)	32 (13.2)	242 (25.3)
	>71	36 (62.1)	1 (1.7)	16 (27.6)	5 (8.6)	58 (6.1)
Total		487 (51)	189 (19.8)	209 (21.9)	70 (7.3)	942^a^ (98.6)

^a^A total of 13 respondents did not answer regarding age group.

#### Staff Characteristics

In total, 521 staff responses were received (Table 2), with a response rate of 12.3% (521/4234). The largest number of responses by profession was from Allied Health Professionals (155/521, 29.8%). The largest number of responses by department was from Community Mental Health (188/521, 36.1%). Most staff responses were completed following VCs with a patient for whom staff members had 1-3 previous contacts. Staff data, such as patient data, are episodes rather than individuals.

**Table 2:** Staff respondents, showing profession and department (N=521).

Characteristics		Participants, n (%)
**Profession**		
	Nurse	81 (15.5)
	Psychologist	117 (22.5)
	AHP^a^	155 (29.8)
	Physician	61 (11.7)
	Other	107 (20.5)
**Department**		
	ACS^b^ inpatient	17 (3.3)
	ACS community	114 (21.9)
	Community Mental Health	188 (36.1)
	Mental health inpatient	4 (0.8)
	CAMHS^c^	127 (24.4)
	Children’s Services	56 (10.7)
	Complex care and dementia	10 (1.9)
	AMH^d^ and learning disabilities	5 (1)

^a^AHP: Allied Health Professionals.

^b^ACS: Adult Community Services (eg, podiatry, spinal, physical, and rehabilitation).

^c^CAMHS: Child and Adolescent Mental Health Services.

^d^AMH: Adult Mental Health.

#### Patient Overall Experience and Future Intention

Most patients (890/955, 93.2%) reported having a *good* (210/955, 22%) or *very good* (680/955, 71.2%) overall experience with VC. A small number of patients had a *poor* (13/955, 1.4%) or *very poor* (17/955, 1.8%) experience. Future intention could also be seen as a measure of satisfaction with VC—9 out of 10 patients were *very likely* (704/955, 73.7%) or *somewhat likely* (176/955, 18.4%) to choose a VC in the future. Very few patients (28/955, 2.9%) suggested they were *somewhat unlikely* (17/955, 1.8%) or *very unlikely* (11/955, 1.2%) to use VCs in the future. Within the results, we were able to look at two aspects of overall satisfaction: satisfaction with the technology (video and sound) and satisfaction with the communication (more related to the clinician’s performance in this situation).

#### Patient and Staff Technical Satisfaction

Three-quarters of the patients reported having a *very positive* experience with sound and video quality (732/955, 76.6% and 728/955, 76.2%, respectively). When combined, 67.6% (646/955) had a very positive experience with both video and sound (Table 3). Three-quarters of staff also reported a *positive* or *very positive* experience with sound and video quality (411/521, 78.9% and 399/521, 76.5%, respectively).

**Table 3:** The 4 indicators of patient satisfaction with video consultations shown by patient age and device used, showing *P* values from chi-square test (N=955).

Characteristic		Positive overall	Would choose VC^a^ again	Positive about technology	Positive about communication	Total patients
**Age (years)**						
	<18, n (%)	153 (93.9)	146 (89.6)	94 (57.7)	137 (84)	163 (17.3)
	18-30, n (%)	133 (91.1)	134 (91.8)	92 (63)	131 (89.7)	146 (15.5)
	31-50, n (%)	316 (94.9)	313 (94)	240 (72.1)	290 (87.1)	333 (35.4)
	51-71, n (%)	224 (92.6)	225 (93)	168 (69.4)	200 (82.6)	242 (25.7)
	>71, n (%)	55 (94.8)	53 (91.4)	46 (79.3)	53 (91.4)	58 (6.2)
	Total, n (%)	881 (93.5)	871 (92.5)	640 (67.9)	811 (86.1)	942^b^ (100)
	*P* value	.55	.49	.003^c^	.18	N/A ^d^
	Chi-square (*df*)	3.0 (4)	3.4 (4)	15.8 (4)	6.2 (4)	N/A
**Device**						
	Laptop, n (%)	445 (91.4)	441 (90.6)	307 (63)	407 (83.6)	487 (51)
	Mobile phone, n (%)	184 (97.4)	179 (94.7)	147 (77.8)	168 (88.9)	189 (19.8)
	Tablet, n (%)	200 (95.7)	195 (93.3)	152 (72.7)	183 (87.6)	209 (21.9)
	Other, n (%)	61 (87.1)	65 (92.9)	40 (57.1)	60 (85.7)	70 (7.3)
	Total, n (%)	890 (93.2)	880 (92.1)	646 (67.6)	818 (85.7)	955 (100)
	*P* value	.003^c^	.28	<.001^c^	.27	N/A
	Chi-square (*df*)	13.8 (3)	3.9 (3)	19.6 (3)	3.9 (3)	N/A

^a^VC: video consultation.

^b^A total of 13 missing ages.

^c^*P*<.05.

^d^N/A: not available.

#### Patient and Staff Satisfaction With Communication

Most patients felt that they had been listened to and understood (904/955, 94.7%), had had their needs met (860/955, 90.1%), and had been able to communicate everything they wanted (848/955, 88.8%). Overall, 85.7% (818/955) of the patients rated all 3 aspects positively (Table 3).

Most staff members (419/521, 80.4%) felt able to communicate everything they wanted to, although satisfaction was slightly lower than that of patients (419/521, 80.4% vs 848/955, 88.8%; χ^2^_1_=19.4; *P*<.001). Staff perceptions of patients feeling their needs were met were generally positive, with 57% (297/521) and 29.8% (155/521) responding *yes* and *yes partially*, respectively. Only 2% (19/955) of patients said their needs were not met, whereas 11.1% (58/521) of staff believed that patients felt their needs were not met, suggesting an apparent discrepancy.

#### Association Between Patient Satisfaction With Technology and Communication (Combined as Above)

Patient satisfaction with communication was very strongly positively associated with satisfaction with technical performance (χ^2^_1_=104.0; *P*<.001; Table 4). Only 4.3% (41/955) of patients were less satisfied with the communication despite being satisfied with the technology. Conversely, 22.3% (213/955) of patients remained positive about the communication despite being less positive about the technology.

**Table 4:** Cross-tabulation of patients being positive about technology with patients being positive about communication (N=955).

Positive about technology	Positive about communication, n (%)		Total, n (%)
	No	Yes	
No	96 (70.1)	213 (26)	309 (32.4)
Yes	41 (29.9)	605 (74)	646 (67.6)
Total	137 (100)	818 (100)	955 (100)

#### Patient Independence and Accessibility

Most patients (806/955, 84.4%) stated that they could access their VC alone and that it was *very easy* or *easy* to join the VC call (849/955, 88.9%). However, more respondents aged >71 years reported needing help (35/58, 60%), and fewer reported it easy to join compared with those aged <31 years (40/58, 69% vs 286/309, 92.6%; χ^2^_3_>=28.6; *P*<.001).

#### Influence of Patient Age and Device Used on Outcome

The relationship between device used, age, and satisfaction with technology and the VC was complex. On the one hand, older patients were more positive about the technical experience despite being more likely to need help accessing the VC and being less likely to find accessing the VC easy. Mobile users were also more positive (Table 3), and older adults were less likely to be mobile users (Table 1).

#### Staff Experience With Managing and Joining the Call

Most staff respondents reported a *positive* or *very positive* experience when managing and joining the VC (466/521, 89.4%). A smaller number of staff responses indicated a *negative* or *very negative* experience (49/521, 9.4%).

#### Patient and Staff Perceived Savings

Two-thirds of patients reported a perceived saving in time (662/955, 69.3%), with more than half of respondents also reporting a perceived saving in money (544/955, 57%). There was no difference by age. Staff respondents most commonly identified carbon savings (380/521, 72.9%) followed by time (373/521, 71.6%). Over one-third of staff reported saving money (187/521, 35.9%). Just below one-quarter of staff respondents reported a perceived saving on missed appointments or *did not attends* (DNAs; 128/521, 24.6%). Approximately 24% (125/521) reported *other* unspecified savings. Savings are explored in greater depth in the qualitative analysis below.

#### Patient Versus Staff Perception

Overall, there appears to be good concordance between staff and patient feedback, with similar benefits noted for time and money savings. Mann–Whitney *U* tests demonstrated no significant difference between 521 staff and 955 patient ratings of video (*P*=.15) or sound quality (*P*=.77). However, significant differences between staff and patient responses were identified when reviewing whether patients had been able to communicate everything needed and felt their needs were met. On both occasions, staff responded more negatively than patients (*P*<.001; *P*=.02). This could suggest that staff overestimated patient dissatisfaction with VC outcomes or were not aware of patient experiences. This is a useful finding, which was reported back to stakeholders at the Trust; however, limitations in the survey (as discussed in Multimedia Appendix 1) must also be considered.

### Qualitative Results

#### Number of Comments

Overall, 13.9% (133/955) of patients made 1384 free-text comments in response to one or more of the 16 questions (Multimedia Appendix 1). Patients who rated their overall experience as good or very good were much less likely to comment (105/890, 11.8% vs 28/65, 43.1%; *P*<.001). Two-thirds (350/521, 67.2%) of staff made 528 free-text comments in response to one or more of the 9 questions asked.

#### Overall Themes

##### Overview

Inductive thematic analysis of free-text responses identified three main themes: barriers, benefits, and suggested improvements. Although the overarching themes were the same based on staff and patient analysis, there was some variation in initial codes between the 2 groups, as discussed in the narrative below and shown in Table 5. Unique identifiers are used for each quote.

**Table 5:** Qualitative patient feedback themes, subthemes, and codes.

Theme and subtheme		Codes
**Barriers**		
	Technological issues	Equipment, sound or video issues, difficult to communicate, connectivity issues, sound quality, video quality, joining issues (SP^a^) Impaired therapeutic flow, limited support, poorer-quality interactions, increased staff stress (S^b^)
	Quality of patient information and administrative support	Jargon, accuracy, complexity of language (P^c^) Lack of technical support, human error, patient struggles with VCs^d^ and joining (SP)
	Accessibility and suitability concerns	Lack of suitable or compatible devices and up-to-date browsers, support required (SP) Widening inequalities, difficult to risk-assess, no hands-on care, suitability of certain conditions including the following (S): Hearing-impaired (SP) Children and attentional issues (S)
	Time, resource, and cost concerns	Increased personal cost for staff, increased staff time, more DNAs^e^ (S)
**Benefits**		
	Reduced anxiety and stress	Comfort, face-to-face element, relaxing and anxiety-reducing, safer, patients better supported, family members present (SP) Ability to open up more (S)
	Continued service delivery	Allows examination, facilitates contact with patients and staff, higher-quality appointments, nonverbal cues, safety, continued service (S)
	Perceived savings	Travel, money, time, environment, work hours, arranging lifts, childcare (SP)
	Enhanced accessibility	Increased access, affordability of attending, comfort, childcare, arranging lifts (SP) Fatigue (P)
**Suggested improvements**		
	Information and support improvements	Notify if appointments are running late (P) Allow trial VC, provide reminders, simplify or improve patient information, simplify sign-in process, device advice (SP) Camera positioning guidance (S)
	Technological improvements	Turn off music, allow photo upload before appointment, support use in other browsers, expand character limit, allow document editing (P) Shared interactive whiteboard, resources or activities, background blur for privacy, allow control by patient (S)

^a^Codes present in staff and patient data.

^b^Codes that resulted only from staff data.

^c^Codes that resulted only from patient data.

^d^VC: video consultation.

^e^DNA: did not attend.

##### Barriers

The participants related barriers from technological difficulties, quality of patient information, and concerns about accessibility or suitability of using VC.

###### Technology

Many patients identified concerns of connectivity—“platform glitches” [Patient 94]—or experienced delays between video and sound:

The picture kept freezing and pixelating.

Patient 741

The video quality is so poor it’s really hard to get much done.

Patient 332

In some cases, technical difficulties meant patients felt that their “needs were not met” [Patient 305]. Similarly, some clinicians reported having to “resort to a telephone” [Staff 14] to supplement VC audio. Some staff members reported that VCs had “a very negative effect on the quality of...therapy we can deliver” [Staff 28] and “limit...the complexity of the conversation” [Staff 29], making it “hard to pick up on body language” [Staff 144]. For some staff members, impairments caused by technological issues were seen as “detrimental to patient care” [Staff 90], “frustrating” [Staff 197, Staff 409, and Staff 439], and “stressful” [Staff 90]. It is possible that the connectivity issues seen in this study related to the geographical character of the region of Cornwall, with staff reporting the following:

The general Cornish bandwidth is the obstruction here.

Staff 118

However, staff reported other platforms seemed to encounter fewer issues, suggesting the platform “needs to be improved substantially and quickly” [Staff 22].

###### Quality of Patient Information and Administrative Support

The quality of patient information, particularly joining instructions, was repeatedly called into question by both patients and staff. A patient described the joining process as “stupidly complex” [Patient 52]. Others described being “directed to a troubleshooting, jargon filled, suggestions page” [Patient 95]. The accuracy of the joining instructions was also questioned:

The link doesn’t open as the instructions [suggest].

Patient 236

Although a video version of the information was provided, this was described by the participants as in need of further development and refinement to ensure inclusivity, particularly for “deaf patients” [Patient 582]. Similarly, staff felt that the “main issues” [Staff 75] for patients involved “logging on” [Staff 71] and that “the process of joining has lots of information to process” [Staff 75]. The consequence being “patients [take] a while to get into the appointment [as] the process [is] complicated” [Staff 150]. Some clinicians needed to “telephone and talk [the patient] through logging on” [Staff 286].

###### Accessibility

Access to relevant devices, browsers, digital skills, and confidence were also described as problematic by some participants:

Unfortunately I was not up to date with technology.

Patient 319

Some patients reported needing to download alternative browsers or borrow other people’s devices because of “outdated” [Patient 671] models. Staff also suggested the following:

It can be hard to engage those with limited IT equipment.

Staff 106

Similar to the quantitative findings outlined above, some participants reported needing help from family members or friends as “without [them] it would have been impossible” [Patient 540]. Another “96 year old had to pay for a carer to be present [and] 2 hours of IT help from someone else” [Patient 47], raising further questions and concerns.

###### Suitability Concerns

Although patient concerns focused mainly on technology and digital exclusion, staff had additional concerns about suitability based on patient illness or requirement. Some staff members felt that VCs were exacerbating “health inequalities” for individuals with learning disabilities or living in residential homes as patients were “often excluded from the review” [Staff 202] as computers were often located “in an office” [Staff 202]. This concern was echoed, as “clients with learning disabilities” [Staff 15] often “need reasonable adjustments to be facilitated to communicate” [Staff 15]. The usefulness of VCs for dementia services was also queried, with the “screen [removing] sensory aspects and visual clues” [Staff 130]. VCs were also seen as unsuitable for dysphagia, where “a hands on approach is required to closely look, listen and feel as the person eats and drinks” [Staff 25]. Patient conditions that may impede VC success as suggested by staff respondents also included “cognitive, speech, language, fatigue, concentration, need for physical...assessments, environmental assessments, safety” [Staff 23]. Other areas described as problematic included family therapy, where the “family had to sit side by side, so parents couldn’t see their child’s facial expressions” [Staff 278], and VCs with children generally, particularly with attentional needs, which “meant [the] session was longer” [Staff 136] and fewer tasks were achieved than face to face. Patient safeguarding and “environmental assessments” [Staff 23] also appeared to be a key issue for clinicians—“home visits remain hugely important to gather information to ensure patient safety,” [Staff 130] providing more information “such as how a person may be living and identify self-neglect, declines in functional skills or poor medication management” [Staff 130]. Staff also suggested some conversations may “be very challenging” [Staff 202] over VC, such as discussing “the risk of dying over the internet and not in person” [Staff 202], as some respondents felt “discussing end of life care” [Staff 29] over video carried “an increased risk of missing cues” [Staff 29]. Patients raised concerns for people with hearing impairments as “face to face is easier” [Patient 457] for lip readers because of video or sound delays and character limitations in the VC chat function.

###### Time, Resource, and Cost Ineffectiveness 

A minority of staff members reported concerns of time, resource, and cost ineffectiveness as, although VCs “saved a few minutes walking to the clinic and tidying the room for the assessment,” [Staff 169] they could also “add an extra appointment” [Staff 169] when a face-to-face consultation was needed. Some respondents also suggested that VCs were longer owing to a “longer explanation time” [Staff 279] to talk patients through the process of how to log on, as described above. Technological issues also affected duration, with patients “having to change rooms” [Staff 198], “change to telephone” [Staff 203], or “re-join” [Staff 19] after disconnection. Furthermore, staff felt VCs “take quite a bit of preparation prior to the consultation” [Staff 23], particularly in psychological services where staff may “need to make electronic versions of therapy resources” [Staff 6]. Although 24.6% (128/521) of staff reported reduced DNAs numerically, some staff members suggested DNAs had increased:

I’ve had more DNAs than when most of my visits were by car.

Staff 413

However, only 3 such comments were made. Interestingly, patients did not report time or resource ineffectiveness and generally reported savings, as described below.

Interestingly, none of the patients described the therapeutic relationship or quality of care delivered by individual health care professionals as a barrier or limitation of VC use.

##### Benefits

###### Overview

The participants identified benefits of continued high-quality service delivery, including reduced anxiety and stress, perceived savings, and enhanced accessibility. Most patients repeatedly described their VC experience as “fantastic” [Patient 558], “excellent” [Patient 579], “amazing” [Patient 153], “wonderful” [Patient 863], “positive” [Patient 600], and “useful” [Patient 667]*.* The participants also appeared to appreciate being able to have “family members join the appointment” [Patient 15].

###### Continued High-Quality Service Delivery 

For staff, VCs allowed them to “continue to provide care” [Staff 47] and “maintain contact and show patients that we are here, that we are holding them in mind and we are motivated to help” [Staff 24]. Clinicians noted that, without VCs, provision would be “even more reduced” [Staff 47] and that services such as “psychotherapy” [Staff 160] did not need to go “on hold” [Staff 160]. Staff who were “shielding” [Staff 356] also managed to maintain workloads and provide care for “shielding patients” [Staff 394]. Some clinicians reported good “depth of therapy work,” which was “less tiring than therapy by telephone” [Staff 60].

###### Reduced Anxiety and Stress 

Both patients and staff felt that VCs were “far less stressful” [Patient 796] than face-to-face consultations. Several patients reported feeling “more relaxed” [Patient 563] and less “rushed” [Patient 392] as a result of avoiding certain stressors, including arranging transport, arriving on time, finding and paying for parking, and traffic. Reduced anxiety and stress were also reported among children, particularly in children who experienced anxiety about appointments or leaving the house. Clinicians noted that child assessments could be supported by the presence of teaching staff in addition to parents alone and noted the following for adults with learning disabilities:

Distant parents [were] able to join the review, whereas they wouldn’t have previously.

Staff 202

Staff also reported that VCs were “less stressful” [Staff 89] for the clinicians themselves, “increasing my wellbeing” [Staff 64] by “saving stress” [Staff 209] and “anxiety and distress” [Staff 519]. Other staff members suggested that VCs “can actually be more therapeutically productive” [Staff 170] with “improved communication” [Staff 110] and “better clinical contact” [Staff 200]. Patients reported that the removal of “so much stress” [Patient 290] meant that they had “more time to focus on what needs talking through” [Patient 290]. Several participants suggested that, as they “didn’t feel so stressed” [Patient 695] and were in the comfort of their “own home” [Patient 564], they were “able to open up more” [Patient 695], often feeling more “comfortable” [Patient 564] and “relaxed” [Patient 564]. Similarly, for staff, patients receiving care at home was seen as beneficial, such as for a “post-natal mum” who “felt more comfortable in their own home” [Staff 126]. VCs were also seen as reducing “anxiety for patients concerned about face-to-face appointments due to COVID” [Staff 29] and allowed for “concordance” [Staff 58] in patient care.

###### Perceived Savings 

Both patients and staff reported time, monetary, and environmental savings. Some patients reported saving “over £20 in transportation costs” [Patient 98] and being able to now “afford” [Patient 153] an appointment as a result of time and cost savings:

[VCs] have genuinely changed my life...being accessible for my needs and being able to afford an appointment.

Patient 153

Travel and cost savings may be particularly prevalent in “Cornwall,” where it “is always difficult to travel for appointments” [Patient 262]. Many staff members also suggested VCs save “time, money and travelling for all concerned” [Staff 324], also reducing “carbon footprint” [Staff 75] by saving “on travel” [Staff 315], “paper” [Staff 242], and “printing resources” [Staff 261]. Many clinicians reported that they could “see patients more intensively” [Staff 7] and “complete an increased number of appointments in a day” [Staff 19] with “increased capacity as a whole” [Staff 17]. Clients who were often “late due to travel” [Staff 81] were now on time. Clinicians reported saving “90 minutes in the car visiting a patient who is just as happy to be seen by video” [Staff 307]. Some staff members also reported that they “rarely get DNAs” [Staff 444] and that VCs “must have saved [The Trust] a lot of money” [Staff 444].

###### Enhanced Accessibility 

Although digital exclusion was thought to reduce accessibility for some, as outlined above, most free-text responses suggested that VCs facilitated service accessibility in a number of ways. First, owing to certain conditions and reduced mobility, some respondents found “trips to the hospital very tiring and difficult” [Patient 20]. VCs removed this experience. Childcare and employment cost savings were also described, helping increase accessibility. For example, a participant suggested that, for a face-to-face appointment, they “would have been dragging all three kids along for what ended up being something that the video call was able to address” [Patient 863]. The “option of video call” [Patient 335] was also considered “useful” [Patient 335] for those who are employed or “working parents” [Patient 335]. Patients could schedule weekly appointments around their employment, something that is not always possible when relying on face-to-face appointments. Furthermore, a number of patients who were “not able to drive” [Patient 307] or “can’t drive” [Patient 846] described VCs as “much more convenient” [Patient 307] because of enhanced independence and removal of reliance on others. Similarly, staff reported increased accessibility for numerous patients who ordinarily “cannot travel to appointments” [Staff 77], such as those with “mobility issues” [Staff 201]. The lesser time requirement for patients to attend via VC was considered to increase “availability of services to clients with other commitments” [Staff 274], “work engagements” [Staff 153], or “caring responsibilities” [Staff 280].

As a result of the benefits encountered, many patients expressed a strong desire for VCs to be made available beyond the COVID-19 pandemic:

I hope that all of our future appointments will be held this way.

Patient 863

This highlights an important element of patient choice.

##### Suggested Improvements

Finally, staff and patients suggested improvements in two main areas: information and technology.

###### Information Improvements 

Respondent suggestions included the simplification of patient guidance and information, device-specific advice, and suggested device use for optimum VC experiences. For example, some patients reported positioning their “iPad onto the floor, so I could show...my feet and me walking” [Patient 248]. This would have been less feasible if using “a desktop computer” [Patient 248]. Other participants suggested it was “a bit challenging getting [the] camera in position to demonstrate me doing the exercises” [Patient 809]. Therefore, providing device-specific information and recommending particular devices based on service requirements and availability may be beneficial. Patients also requested some method of notification if the clinics were running late.

###### Technology Improvements

Related to platform functionality, patients requested the ability to “turn off the music while sitting in the waiting room” [Patient 388] as it was considered “terrible” [Patient 517] and repetitive by some. Patients also expressed a desire to be able to “upload photos or videos” [Patient 20] to the consultation and “change the mobile camera being used” [Patient 145]. Further platform-related improvements suggested included expanding its functionality to “other browsers” [Patient 342] and having the character limit expanded beyond 200 characters for patients who need to use the text function. Other suggestions made by staff included more interactive screen-sharing capabilities as currently clinicians “cannot see [the] client anymore” [Staff 6] when sharing their screen. Others thought it would be useful if the clinician could “allow the client to take control of a particular programme you are sharing,” [Staff 6] which could help with “engaging children so you could play games together” [Staff 6]. A similar desire was noted for adult cognitive behavioral therapy, with staff requesting a “white board” [Staff 119] to “draw things out on such as CBT formulations” [Staff 52]. The absence of such functionalities meant that diagrams were completed less “collaboratively” [Staff 107] than if patients were “in the room” [Staff 107]. Finally, related to digital skills and confidence, some patients expressed a desire for a “dummy run” [Patient 74] to be made available so that people could familiarize themselves with the technology before their consultation. Interestingly, no suggestions for health care training were proposed by the participants, although this may reflect the questions asked in the feedback survey. For enhanced security, staff requested a “blurred background” [Staff 3] option “to help protect privacy” [Staff 3] and “improve confidentiality” [Staff 375]. This seemed particularly relevant for clinicians who “work with forensic clients” [Staff 3]. An additional improvement would be having the ability to “lock the room once everyone is in” [Staff 8] after experiencing “incidents of other staff joining private, confidential therapy sessions uninvited” [Staff 8].

###### Comparison of Patient and Staff Feedback

Generally, staff and patient responses showed high congruence, as evidenced by the similarity of codes and subsequently combined themes. However, as with the quantitative results, where more staff members responded with concern about patients feeling their needs were met than patients themselves, the qualitative data also demonstrate some evidence of clinicians believing that VCs impaired the therapeutic flow or produced poorer-quality interactions. Patients provided no indication of dissatisfaction with the clinician’s communication, outcomes, or care received other than issues resulting from the technology. Although staff concerns on meeting patients’ needs also commonly resulted from technical issues, generic concerns were also shared on therapeutic quality and missing cues or body language via VC. In addition, patients regarded highly the increased accessibility of health care and found appointments less stressful. The codes on time, resource, and cost ineffectiveness were provided only by staff, whereas patient data strongly supported perceived savings across time, money, travel, and environmental impact. Staff and patients showed similarity in the reported requirement for improved sound and video quality. In addition, patients requested the removal of waiting room music, expansion of character limits, and trial runs, whereas staff requested blurred backgrounds and interactive shared screens. Both patients and staff requested simplified or improved patient information.

### Documented Impact of Routine Feedback on Real-world Practice

Following the initial analysis of the results, the authors presented the above findings to relevant patient and professional stakeholders to provide rapid feedback on barriers and facilitators, possible areas for improvement, and ways to encourage the sustainable use of VCs. The findings were positively received by both clinicians and patients. In particular, clinicians reported underestimating patient satisfaction with VCs and were surprised to see such high levels of satisfaction with the service, particularly patient perceptions of communication quality and feeling their needs were met. The presentations aided in revising clinician perceptions, and some initial changes to VC practice have already been instigated, including the replacement of waiting room music with bird songs and efforts to improve patient information. Thus, the use of routine feedback and its analysis was instrumental in instigating some initial improvements for the use of VCs within the NHS Trust and promoting further conversations around future VC use and improvements. The implementing NHS Trust has now confirmed the procurement of Attend Anywhere for VC service provision to continue beyond COVID-19 restrictions.

## Discussion

### Principal Findings

This research contributes to the existing literature exploring staff and patient experiences with the rapid rollout of VCs for outpatient care during the COVID-19 pandemic [21], with clear implications for policy, practice, and future research. Specifically, this work contributes to the VC evidence base with a considerably larger sample than previous evaluations, in a rural setting and with a focus on VCs rather than telephone. We also provide documentation on the impact of routine feedback. Unlike existing literature mainly focusing on primary care or specific health services, this research explored the use of VCs across outpatient services more broadly, helping address limitations within the existing COVID-19–response literature [4,10].

Most patients rated their VC experience as *good* or *very good*, felt listened to and understood, were able to communicate everything they wanted to, and felt their needs had been met. Many patients reported saving time and money, and >90% (880/955) would likely choose a VC in the future, although it remains unclear whether this resulted from no perceived *alternative* option owing to the global pandemic or from positive patient experiences and related motivations. Further exploration would be beneficial, including analyzing patient experience over time. Staff also generally supported the use of VCs through positive experiences with joining and managing calls, being able to communicate all that was needed, and feeling the patients’ needs had been met, although agreement on the latter issue was not unanimous. Staff also reported savings, mostly in terms of carbon footprint and time. Qualitatively, both staff and patients noted increases in service accessibility and affordability. As a result, this Trust commissioned Attend Anywhere for future (COVID-19 and post–COVID-19) use, and other Trusts should consider making VCs a permanent option beyond pandemic restrictions.

Patients aged ≥71 years (58/955, 6.1%) were the only age group in which a larger percentage of respondents reported needing support accessing their VC than those who were able to access alone. Nevertheless, there was no clear age gradient in satisfaction with VCs. Indeed, older adults were more likely to be positive about the technology than younger people. This may reflect higher expectations among younger people. Although high levels of positive experience were reported across all device types, more users of tablets or mobile devices were positive. The relationship between age, device used, and positive experience with VCs is complex. Research suggests that the small size of mobile phones can pose a barrier for older adults coupled with declining dexterity and vision. Mobile phones are mainly used by older adults for calls and texting [25], whereas tablet computers are more popular for web-based access among older adults than among younger people [26]. Older working age groups may be more likely to prefer technologies they are familiar with at work (often desktops with poor cameras and sound systems), whereas older adults new to computing use tablets as their entry device [27].

Clinical areas of less suitability for VCs were also noted, particularly by staff. Further research is needed to identify when VCs work best, for whom, and in what context. Although Greenhalgh et al [7] have already provided guidance on appropriate and inappropriate use of VCs, a more granular understanding from both a patient and professional perspective may be required. Some limitations were noted for spinal services, neurology, children, attentional issues, and assessing dysphagia. The previous guidance [7] suggested that inappropriate contexts included patients at high risk, patients requiring internal examination, and patients with challenges affecting the ability to use technology. This suggestion is supported by our work, with staff suggesting VC was less appropriate for patients with learning disabilities, communication disorders, fatigue, cognitive issues, or dementia.

Barriers described by staff and patients included technological difficulties, quality of patient information, administrative errors, and accessibility or suitability concerns. Conversely, identified benefits included reduced stress and anxiety for patients and staff, the opportunity to “open up more” for patients as a result of enhanced comfort, cost and time savings, increased sense of affordability, and service accessibility. Finally, the participants suggested a number of improvements, such as simplifying patient information, notifications for late appointments, the ability to turn off waiting room music, a shared interactive whiteboard, blurred backgrounds, and “practice run” opportunities to increase familiarity and digital skill confidence. Other suggested improvements included allowing photo or video upload to the appointment, swapping between cameras used, extending Attend Anywhere to other internet browsers, and expanding the character restrictions of the chat function, particularly important for accessibility of deaf patients. Thus, this study has a number of practical implications. Routinely collecting and responding to feedback is likely to be an integral aspect of service improvement, as demonstrated in this study. Feasible improvements such as those reported here are likely to have important impacts on staff and patient experience.

The need to simplify and improve patient information was highlighted as a key barrier by both staff and patients, and this may be best achieved in co-design with patients. Although the implementation of VCs was a rapid response, actively involving patients and the public and creating digital-related information may improve accessibility, relevance, and understanding. Thus, an implication of this work is an identified need to establish the best practice for rapid co-design when implementation timing is critical. Any future patient information may also include guidance for patients on camera positioning to reduce another barrier identified in this work. Health care services may also benefit from recommending particular devices based on their functionality and service requirements. For example, larger, static screens may be suitable for child therapies or family-based interventions where patients and families need to sit side by side. Alternatively, VCs that include assessment of movement may be better suited to more portable devices such as mobile phones or tablets.

An important consideration for VCs is safeguarding. Bhardwaj et al [13] reported that clinicians were confident in performing safeguarding and risk assessments remotely. Our results indicate otherwise as clinicians reported that home visits were key for patient safeguarding and to allow monitoring of self-neglect, decline in well-being, or poor medication. Therefore, home visits for patients requiring environmental assessments could be prioritized for face-to-face appointments, as could consultations relating to the identified less appropriate contexts. However, this assumes that environmental assessments are not possible via VC, where perhaps a visual tour of the home environment during a VC would suffice. Thus, an alternative implication is guidance for clinicians in this regard. A further concern was raised by clinicians consulting with patients in residential care with respect to widening health inequalities as patients are often unable to attend consultations where computers are housed in staff offices. In addition, some patients in these contexts appeared unable to appropriately position their camera, suggesting the potential for solutions such as affordable telepresence devices or robotics in residential care to facilitate VCs. This could respond to two barriers: (1) patients being excluded from VCs because of equipment in staff offices and (2) challenges regarding appropriate camera positioning.

As the final practical implication, in response to patients noting administrative and human errors, the collaborative development of checklists and supportive training may be beneficial. Trusts could perhaps include on-screen checklists on patient records to ensure that scheduling of VCs is followed by provision of an appropriate link, patient-facing guidance, and setup support. Clinicians may also consider promoting the benefits of teachers or distant relatives attending a VC.

Our study also raises implications for the collection and use of routine feedback. The clinicians in our sample overestimated patient dissatisfaction with VCs. More clinicians than patients also responded negatively to communication quality. A minority of clinicians reported some impairment of therapeutic flow. Our presentation of these results to Trust stakeholders supported this observation, with clinicians surprised about high patient satisfaction. Some clinicians reported avoiding VCs for fear of patient dissatisfaction. Thus, the provision of this routine feedback aided in addressing staff perceptions. It is possible that low staff expectations for VCs somewhat explains the low documented uptake of VCs in comparison with telephone calls in previous research [10,13,14]. When collecting routine feedback, critically considering the purpose is important. For example, Sibley et al [28] recently likened the increasing collection of patient feedback to an “avalanche...with experience now tracked, monitored and measured to an almost obsessive degree” [28]. However, to what end and for what purpose? Reflecting previously acknowledged concerns around the ethics of collecting patient feedback that leads to minimal direct benefit [29,30], Sheard et al [31] suggested all patient feedback tools must be meaningfully usable by those providing frontline care; otherwise, it becomes “unethical to ask patients to provide feedback which will never be taken into account” [31]. Thus, service providers should ensure that routinely collected feedback (including after an Attend Anywhere appointment) is meaningful for both patients and clinicians, serves a beneficial function beyond mandatory feedback collection, and focuses on care delivery aspects that are most important to patients and clinicians. Future research may consider which feedback methods are most effective in encouraging responses, particularly in the new *digital norm*, with staff members supported and empowered in acting upon and responding to feedback received.

### Limitations

The first limitation of this research is the reliance on a self-selected sample of individuals who attended or facilitated a VC and chose to provide feedback. Experiences or barriers for those unable or choosing not to use VCs currently remain unknown, as do the experiences of those not providing feedback following their VC. Nevertheless, from this study, we know that hundreds of staff members and patients had a positive experience with VCs. Second, owing to the anonymous nature of the data set, we were unable to identify how many individuals completed the survey on repeated occasions; thus, the results may be skewed by repeat respondents. Third, this research relies on secondary data and the subsequent questions or scales used by the Trust. Although informed by the *1-week implementation guide for NHS Trusts and NHS Foundation Trusts* provided by NHS England and NHS Improvement [23], the survey had some limitations—some of the questions were poorly worded, and the questions were not directly comparable between staff and patient questionnaires (a more detailed discussion is provided in Multimedia Appendix 1). Furthermore, to the researchers’ knowledge, the survey was not created in co-design with all relevant stakeholders, including patients and carers. Therefore, the questions asked may not reflect the most important aspects of the VC experience for patients.

Future research could explore challenges and barriers for excluded patients, particularly those considered seldom heard or marginalized in the context of digital health [32]. Murphy et al [12] noted previously that digital consultations increase access for those with information technology skills but may reinforce existing health inequalities. There is an important balance required between acknowledging increased accessibility for some patient groups who may encounter difficulties accessing face-to-face services and acknowledging reduced accessibility for others. This aspect needs urgent further work and reiterates the importance of patient choice and availability of multiple media to access health and care services.

The nature of this study meant that we were also unable to conclude on a number of additional factors, highlighting the scope for further research. Apparent efficiency savings in service delivery via VC should be explored to assess the impact of VCs on clinician and patient experience. Although intensive work may aid in meeting growing health care requirements, workforce burnout poses a danger. Related to efficiency, further research should look to establish DNAs before and since the implementation of VCs, with almost a quarter of staff reporting less DNAs using VCs than with usual practice. This is an interesting result, and further work could explore the reasons for the reductions in DNAs compared with in-person consultations. Although the results suggest a reduction in DNAs generally, 3 staff comments suggested increases. It would be interesting to explore if DNAs that do occur are linked to patient inability to access VCs or lack of confidence with technology. Other implications for future research include a need to identify the mechanisms responsible for the positive patient experiences and high levels of future VC use intentions, as demonstrated by the Trust in this research. By doing so, other Trusts and health care services can engage with acknowledged areas of best practice. Economic evaluations that incorporate clinician, patient, and environmental savings may also be beneficial, although it is important to emphasize that potential cost savings should not take precedence over patient safety, quality of care, and stakeholder experience.

In addition, suggestions made in this research that VCs improve “affordability” of appointments and comfort in sharing personal or clinical information are important areas of future interest. Future research questions could include how and in what ways have VCs affected patient accessibility? Similarly, how, if at all, do VCs affect the therapeutic relationship? Future research could compare patient satisfaction with more conventional face-to-face consultations or other VC platforms. In addition, the range and type of consultations available to patients are currently limited and expressed satisfaction may reflect a lack of choice or alternatives. Future research may review patient experiences over time, particularly during times of heightened and reduced COVID-19 restrictions.

### Comparison With Previous Work

Generally, our results continue to demonstrate positive experiences for staff and patients with VCs during the pandemic [4,9,10,16], furthering previous work with smaller samples and narrower focus. For example, congruent with Gilbert et al [4], we found that positively perceived aspects of VCs included reduced travel times and reduced impact of travel on symptoms such as fatigue [4]. Other similarities with previous work include low confidence reported among some participants [4] and the negative impact of technological limitations and difficulties on patient experience [4,9,12,13]. Findings from this research regarding age differences in independent use and family involvement are also congruent with other research [12,33]. However, given the difficulties that many older adults have in traveling to outpatient clinics [34] and the largely high acceptability of VC use reported in this study for older adults, no quick assumptions should be made about the unsuitability of VC for older adults.

Areas of divergence from the existing literature include patients reporting higher levels of satisfaction and willingness to use VCs in the future than in previous work [4]. Previous feedback was collected within an entirely orthopedic service, which could suggest that greater satisfaction and use intentions are seen here owing to the variety of services included, which may better translate to VC than orthopedics as perhaps a more *hands-on* service. However, this would need further exploration as survey limitations impair our understanding of exactly which service patient respondents accessed. Other contributions of this research include the identification of additional benefits, including enhanced comfort and subsequent ability to “open up more.” This contrasts with the results of Liberati et al [14], who reported impairments to depth of conversation and relational quality via remote means. Although this paper reports on a larger sample, Liberati et al [14] also reported on qualitative interviews. Therefore, this incongruence in results is worth exploring further, perhaps across specific psychological therapy services. In contrast to Isautier et al [9], who suggested that telehealth limitations included poorer quality of communication, our results suggest that most patients were satisfied with the VC aspects related to communication, with combined technical satisfaction being lower, congruent with Kayser et al [10]. In addition, in this study, we provide further insight into the influence of patient age and device used in predicting overall VC experience, with implications for targeted consultations in the future. This research also provides interesting insights into both staff and patients reporting an increased sense of accessibility and patient perception of enhanced affordability. Therefore, our work contributes to furthering previous research [4,9-17] that reported on small sample sizes and generally single-service focus, whereas we report on a comparatively large sample across outpatient services in a rural and older adult setting [18,20].

### Conclusions

In conclusion, most NHS staff members and patients reported positive experiences with VCs for outpatient care in a rural, older adult, and deprived setting. Patients often felt listened to, able to communicate their needs, and understood, and staff and patients noted resource savings and enhanced accessibility. However, some barriers identified, such as technological difficulties, accessibility of patient information, and accessibility or suitability concerns, require further attention if the potential benefits of VCs are to be realized and their use is to be sustained. The implications of this research include the implementation of patient-suggested improvements, including trial calls, turning music off, facilitating photo uploads, expanding written character limit, and supporting VCs on other browsers. Future work may explore the accessibility and experience of patients excluded from this study through lack of VC access. In addition, this study demonstrated the real-world impact of routine feedback and raises further discussion on the future use of routine staff and patient experience data.

## Appendix

Multimedia Appendix 1 Survey questions and response options.

## Abbreviations

DNA: did not attend
NHS: National Health Service
VC: video consultation
