# Supplementary material for: Exploring Patient and Staff Experiences With Video Consultations During COVID-19 in an English Outpatient Care Setting: Secondary Data Analysis of Routinely Collected Feedback Data
Source: JMIR Form Res. 2022 Mar 21;6(3):e30486. doi: 10.2196/30486 (PMC8989384; doi:10.2196/30486)
Supplement: Multimedia Appendix 1 [file formative_v6i3e30486_app1.docx]

**Appendices**

**Appendix 1. Survey Questions and Response Options**

Each question beginning with ‘COMMENT’ allowed for a free-text response. Patient survey is displayed first followed by staff survey.

**Patients**

1. Please tick this box if you do not wish your comments to be made public
2. Service Line
3. Department
4. Team
5. Overall, how was your experience of our service?

very poor, poor, neither good nor poor, good, very good, don't know

1. COMMENTS: Overall, how was your experience of our service?...
2. How easy was it to join the video appointment?

very difficult, difficult, neither easy or difficult, easy, very easy

1. COMMENTS: How easy was it to join the video appointment?...
2. Joining the call

very negative, negative, unsure, very positive

1. COMMENTS: Joining the call...
2. Sound quality

very negative, negative, unsure, very positive

1. COMMENTS: Sound quality...
2. Video quality

very negative, negative, unsure, very positive

1. COMMENTS: Video quality...
2. Being able to communicate everything you wanted to

very negative, negative, unsure, very positive

1. COMMENTS: Being able to communicate everything you wanted to...
2. Feeling listened to and understood?

very negative, negative, unsure, very positive

1. COMMENTS: Feeling listened to and understood?...
2. Feeling your needs were met

very negative, negative, unsure, very positive

1. COMMENTS: Feeling your needs were met...
2. How likely are you to choose a video appointment as an option in your future care?

very unlikely, somewhat unlikely, neither likely nor unlikely, somewhat likely, very likely

1. COMMENTS: How likely are you to choose a video appointment as an option...
2. Were you able to access this video appointment by yourself?

yes, no, someone helped me

1. COMMENTS: Were you able to access this video appointment by yourself?...
2. What type of device did you use for your video appointment?

laptop, mobile, tablet, other

1. COMMENTS: What type of device did you use for your video appointment?...
2. Which internet browser did you use for your video appointment?

chrome, safari, other, not sure

1. COMMENTS: Which internet browser did you use for your video appointment...
2. What is your age?

under 18 yrs, 19-30, 31-50, 51-71, 71 yrs +

1. COMMENTS: What is your age?...
2. COMMENTS: What specialty or service was your Video Appointment for?...
3. Has using this service helped to save you either time, money or other benefits? eg, travel fares, fuel, parking, lost earnings,

Time, money, other

1. COMMENTS: Has using this service helped to save you either time, money...
2. COMMENTS: We are constantly looking at ways at improving our service. ...

**Staff**

1. Service Line
2. Department
3. Team
4. Channel
5. Profession:
6. Is using the video appointment system saving you any of the following: time, money, DNAs, carbon footprint, other 2
7. COMMENTS: Is using the video appointment system saving you any of the following: time, money, DNAs, carbon footprint, other
8. Have you met this patient face to face previously?
9. COMMENTS: Have you met this patient face to face previously?...
10. How many contacts have you had with this patient:
11. COMMENTS: How many contacts have you had with this patient:...
12. Managing and joining the call
13. COMMENTS: Managing and joining the call...
14. Sound quality
15. COMMENTS: Sound quality...
16. Video quality
17. COMMENTS: Video quality...
18. Being able to communicate everything you wanted
19. COMMENTS: Being able to communicate everything you wanted...
20. Did you feel the patient was able to communicate everything and had all of their needs met?
21. COMMENTS: Did you feel the patient was able to communicate everything ..
22. COMMENTS: If there were issues please say how the experience could be improved

**Commentary on limitation in the survey**

While the survey used has provided some useful and insightful results with important implications, the survey holds a number of limitations.

The questions and responses were not entirely comparable between staff and patients. For example, while the patient survey asked about being able to communicate and having needs met separately, the staff survey asked both questions in one.

The wording of some questions could have been much improved, for example, in the staff survey queries if the ‘patient was able to communicate everything’ and ‘had all of their needs met’ in one question. Combining two questions into one is poor practice and limits clarity in the response. Additionally, patients are asked both to provide an age category they fit further to their exact age.

The patients were not able to provide accurate or complete results for the Service Line accessed, and following this evaluation the survey was improved to collect this data from the Waiting Room instead, ie. which service ‘waiting room’ the patient was attending.

The survey did not allow for understanding of repeat visits, nor did it collect data on demographics of the patients or staff completing the survey. This does limit understanding of the sample.

The surveys also suffered from issues in the response options, such as poorly planned scales and missing options (e.g. very negative, negative, unsure, very positive).
